# Correction to: Exogenous Adenosine Antagonizes Excitatory Amino Acid Toxicity in Primary Astrocytes

**DOI:** 10.1007/s10571-024-01490-5

**Published:** 2024-07-11

**Authors:** Yingjiao Liu, Shifeng Chu, Yaomei Hu, Songwei Yang, Xun Li, Qinglian Zheng, Qidi Ai, Siyu Ren, Huiqin Wang, Limin Gong, Xin Xu, Nai-Hong Chen

**Affiliations:** 1 College of Pharmacy, Hunan University of Chinese Medicine & Hunan Engineering Technology Center of Standardization and Function of Chinese Herbal Decoction Pieces, Changsha, 410208 China; 2 State Key Laboratory of Bioactive Substances and Functions of Natural Medicines, Institute of Materia Medica & Neuroscience Center, Chinese Academy of Medical Sciences and Peking Union Medical College, Beijing, 100050 China; 3 Institute for Brain Research and Rehabilitation, South China Normal University, Guangzhou, 510631 China

**Correction to: Cellular and Molecular Neurobiology (2021) 41:687–704** 10.1007/s10571-020-00876-5

The original version of this article unfortunately contained errors in Fig. 3A and a typo in funding section.

In Fig. 3A, the images of Model group and Ade + SCH58261 group are incorrect. The correct complete Fig. 3 is presented here:

**Fig. 3:**
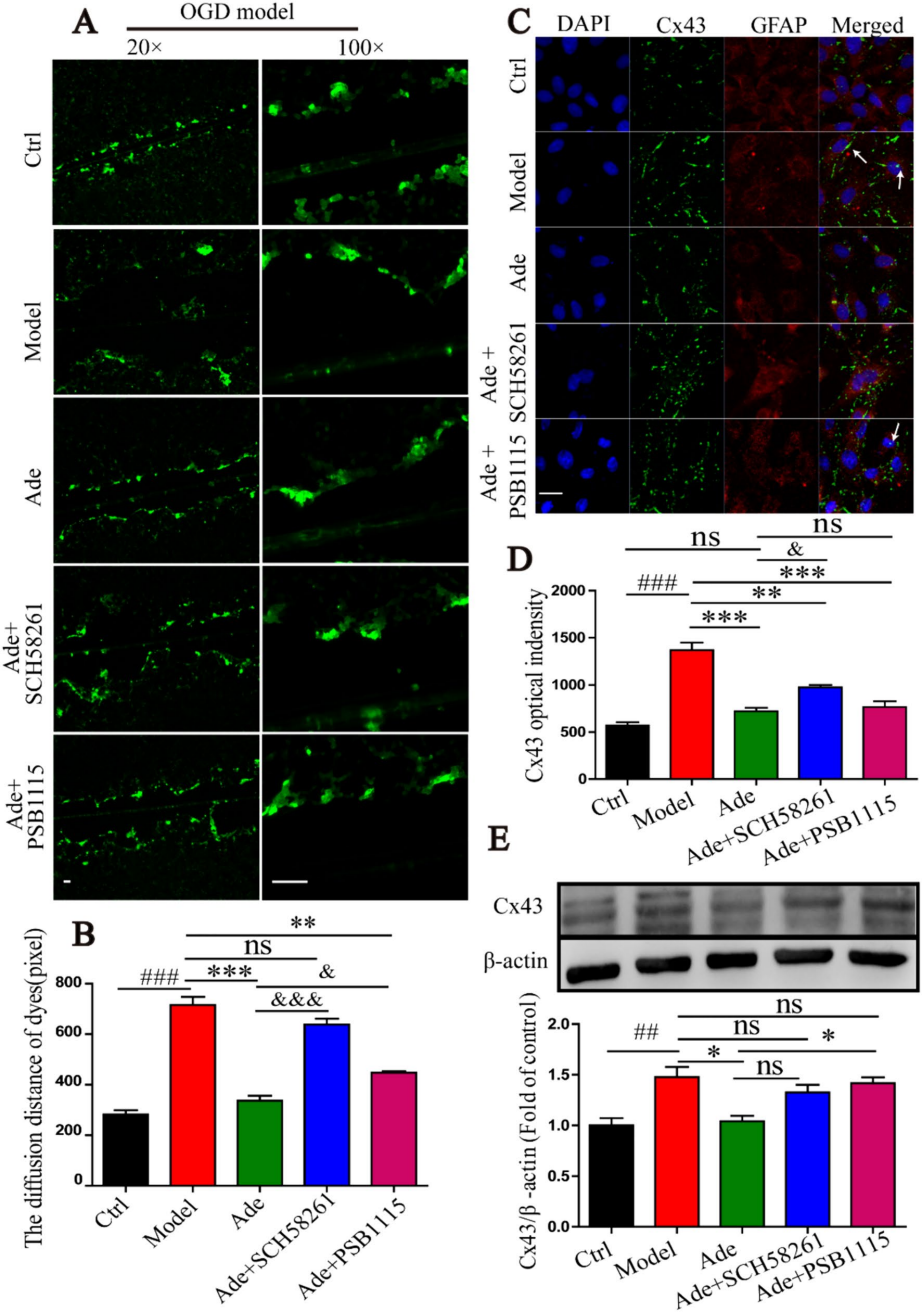
Effects of gap junction intercellular communication in the OGD/R model. **a, b** Cells were subjected to a scrape loading/dye transfer assay using Lucifer yellow. Dye diffusion distance was detected by Image J 5.0 software (bar = 400 μm). **c** Cx43 expression in primary cultured astrocytes in the OGD/R by immunofluorescence technology (bar = 20 μm). The white arrow shows some of the Cx43 transport to the nucleus. Gap junction intercellular communication (GJIC) was inhibited by adenosine. SCH58261 attenuated the ability of adenosine to inhibit gap junction communication. **d** Quantitative analysis of Cx43 optical intensity by image-pro plus 6.0 software. **e** Western blotting and quantitative analysis of the level of Cx43. β-actin served as the internal control, taking the ratio of the gray level of the Cx43 to the β-actin band. All the results were quantifed and were the mean ± SD, n = 3 (independent experiments performed in triplicate). ^###^P < 0.001, ^##^P < 0.01 vs ctrl group. ***P < 0.001, **P < 0.01, *P < 0.05 vs model group. ^&&&^P < 0.001, ^&&^P < 0.01, ^&^P < 0.05 vs ade group. ^ns^P > 0.05 show no statistical difference between the two groups

Also, in Funding section, the word “Disciple” would have been “discipline”.

The original article has been corrected.

